# The evolution of global health teaching in undergraduate medical curricula

**DOI:** 10.1186/1744-8603-8-35

**Published:** 2012-11-13

**Authors:** Mike Rowson, Abi Smith, Rob Hughes, Oliver Johnson, Arti Maini, Sophie Martin, Fred Martineau, J Jaime Miranda, Vicki Pollit, Rae Wake, Chris Willott, John S Yudkin

**Affiliations:** 1 UCL Institute for Global Health, 30 Guilford Street, London, UK; 2 Women’s Health, Southmead Hosptial, Westbury on Trym, Bristol, BS10 5NB, United Kingdom; 3 UK Department for International Development, 1 Palace Street, London, UK; 4 King’s Centre for Global Health, King’s College London, London, UK; 5 NHS Ealing, London, UK; 6 Independent consultant, London, UK; 7 London School of Hygiene and Tropical Medicine, Keppel Street, London, UK; 8 CRONICAS Centro de Excelencia en Enfermedades Crónicas and Department of Medicine, School of Medicine, Universidad Peruana Cayetano Heredia, Lima, Peru; 9 National Clinical Guideline Centre, Royal College of Physicians, London, UK; 10 Division of Global Health and Human Rights, Massachusetts General Hospital, Boston, MA, USA; 11 Juba Teaching Hospital, Juba, South Sudan; 12 University College London, Gower Street, London, WC1E 6BT, UK

**Keywords:** Global health, International health, Medical education, Undergraduate, Curriculum

## Abstract

**Background:**

Since the early 1990s there has been a burgeoning interest in global health teaching in undergraduate medical curricula. In this article we trace the evolution of this teaching and present recommendations for how the discipline might develop in future years.

**Discussion:**

Undergraduate global health teaching has seen a marked growth over the past ten years, partly as a response to student demand and partly due to increasing globalization, cross-border movement of pathogens and international migration of health care workers. This teaching has many different strands and types in terms of topic focus, disciplinary background, the point in medical studies in which it is taught and whether it is compulsory or optional.

We carried out a survey of medical schools across the world in an effort to analyse their teaching of global health. Results indicate that this teaching is rising in prominence, particularly through global health elective/exchange programmes and increasing teaching of subjects such as globalization and health and international comparison of health systems. Our findings indicate that global health teaching is moving away from its previous focus on tropical medicine towards issues of more global relevance.

We suggest that there are three types of doctor who may wish to work in global health – the ‘globalised doctor’, ‘humanitarian doctor’ and ‘policy doctor’ – and that each of these three types will require different teaching in order to meet the required competencies. This teaching needs to be inserted into medical curricula in different ways, notably into core curricula, a special overseas doctor track, optional student selected components, elective programmes, optional intercalated degrees and postgraduate study.

**Summary:**

We argue that teaching of global health in undergraduate medical curricula must respond to changing understandings of the term global health. In particular it must be taught from the perspective of more disciplines than just biomedicine, in order to reflect the social, political and economic causes of ill health. In this way global health can provide valuable training for all doctors, whether they choose to remain in their countries of origin or work abroad.

## Background

Global health is a key component of many undergraduate medical curricula; however, there is ongoing debate over the content of teaching in this discipline. In an accompanying article, we discussed some gaps revealed by debates about definitions of global health concerning how we think about and teach the discipline. These gaps include a lack of attention to context-specificity; a prescriptive approach to defining ‘what’ global health is; and lack of reflexivity about the goals of global health.

In this article we examine the evolution of teaching on global health in undergraduate medical curricula in the UK and worldwide, considering trends from the 1990s until the present day. Drawing on our analysis in the previous article we suggest ways in which the teaching of global health to medical students might proceed in order to create doctors with the skills and attributes needed to work in this area. We suggest a number of methods of learning and points of intervention in curricula. While we draw on a wide literature on these issues, the analysis is also based on our experiences of teaching global health at University College London (UCL) where the authors are or have been based, either as staff or students, and where medical students have been taught about global health since 2000.

## Discussion

### Trends in global health teaching

In the early 1990s Bandaranayake suggested that the latter half of the twentieth century had seen ‘a steady decline in the teaching of international health in medical schools’ (p. 360). Bandaranayake defined the traditional scope of international health as ‘any instruction in comparative morbidity or mortality, service provision, demographic change and disease prevalence in non-industrialised developing countries’. His survey of 100 medical schools, undertaken in 1989–90, revealed that 61.4% of schools taught any international health in their curricula, while 26.1% had it listed as a separate subject. Despite this apparently sizeable proportion, he concluded that the topic was not a ‘high priority’ in most institutions [1].

Banadaranyake’s pessimism may have been premature as the turn of the century saw a revitalisation of interest. Dedicated global health courses started to appear in the UK [2], Sweden [3], the Netherlands [4], USA [5], Finland [6], Germany [7], Canada [8], Australia and New Zealand, [9] and Peru [10]. Subsequently a discourse developed on the most appropriate content of these educational experiences. This discussion was and continues to run parallel with student demand for global health education both as a formal component of the curriculum and outside it [11,12].

Globalization has led to an increasing flow of pathogens, information, trade, finance, and people between and within countries worldwide. This global integration means that a focus on a biomedical and national model of international health is outdated; instead health risks should be considered to have ‘global causes and consequences’ [13]. Recent events such as avian and swine influenza have served to highlight the dangers of cross-border infection. The development of cultural competency skills has taken on a heightened importance as health professionals interact with increasingly multicultural communities [14,15]. In addition, health professionals are increasingly migrating for work [16]. These realities necessitate a new paradigm in global health education to equip future doctors for the challenges that practising in a globalised society brings [17].

The shifting nature of global health has sparked an interest among many medical students. The results of this interest have reflected the broad nature of global health and have resulted in a wide variety of programmes from individual study and international electives to comprehensive degrees covering social, economic and political determinants of health [11,18]. The teaching lobbied for by students often combines the traditional approach to learning about the epidemiology of disease in lower income countries with teaching on a broader range of topics implicated in determining health and disease patterns [5,18].

Interdisciplinary collaboration has been necessary for the development of courses in global health because the shift from a purely public health approach to a focus on the underlying determinants of health has required a wider variety of skills and knowledge [18]. However, there still tends to be a focus on biomedical approaches to global health, with more students attending tropical/travel medicine courses than ones focusing on determinants of health [7]. Despite student-led lobbying and guidance from national bodies, for example the General Medical Council (GMC) in the UK [19], there remains a need for greater engagement with the broader determinants of health, including global inequalities and variations in healthcare provision [7].

#### A survey of global health teaching

In 2007, we applied our own test of these trends in a questionnaire to medical schools in different parts of the world. We chose nine topics based on our knowledge of what is usually taught under the rubric of global or international health in medical schools. These topics reflect traditional ‘international health’ issues along with a broader ‘global health’ perspective discussed above. We questioned medical schools on the topics taught, how they were taught, by whom and for how long they had been part of the curriculum. These ‘categories’ were:

● effects of poverty and inequality on health (with an international perspective)

● globalization and health

● international comparison of disease burden

● international comparison of health systems

● international elective and exchange opportunities

● international health and development

● international movement of people

● travel medicine

● tropical medicine

As resources permitted only an electronic survey, we consulted the World Directory of Medical Schools at the Foundation for Advancement of International Medical Education and Research (FAIMER) for email contact details of medical schools in different countries. We attempted to take a selection of medical schools from each country represented in the directory, though this depended on the availability of contact email addresses. Further email addresses were obtained from web-based research, academic links and through medical students who were part of the International Federation of Medical Students’ Associations. Initially one person per medical school was invited to respond, and this person – who may have been either an academic or administrative employee – was asked to identify the most appropriate person to respond to the survey. Up to three e-mail reminders were sent per medical school. A geographical breakdown of respondents by continent is listed in Table 1. Using this methodology, 273 schools were contacted worldwide and responses were received from 64 schools, a response rate of 23.4%.

**Table 1:** Number of responses to survey received per continent

**Continent**	**Number of schools**
Oceania	5
Africa	6
South America	7
North America	8
Asia	13
Europe	25
Total	64

The response rate obtained is within the range of expected rates for the email survey method implemented [20]. Nevertheless, there will be some degree of selection bias in the respondents – conclusions cannot be drawn that are representative of medical education globally. However, it does provide a snapshot of global health teaching in the first decade of this century and hints at some interesting trends.

**Figure 1:**
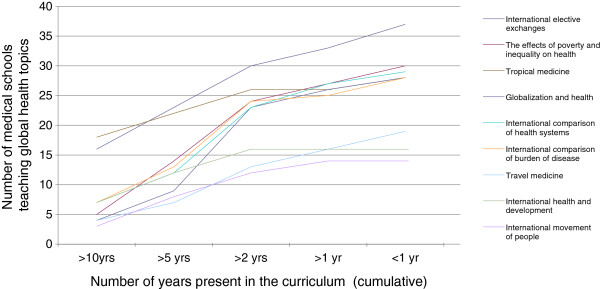
Number of schools teaching global health topics, and length of time they have been taught.

Our results indicate that, firstly, international elective and exchange opportunities appear to be the main gateway for acquiring some interaction with the field of global health. (Table 2) In addition to this, it is encouraging that many of the other topics are addressed to varying degrees by at least some medical schools. Sixty-one (95%) medical schools delivered teaching in at least one of the nine topics surveyed. In contrast to Bandaranayake’s findings (from a survey of different medical schools) our survey indicates new global health topics being added to the curriculum over time (Figure 1) with the balance of what is taught seemingly changing, as indicated by the emerging popularity of globalization and health and international comparison of health systems as topics of study. A number of schools have included elective opportunities and tropical medicine in medical undergraduate curricula for more than ten years.

**Table 2:** Number and percentage of responding medical schools teaching different global health topics

**Topic**	**Number**	**%**
International elective exchanges	37	58%
The effects of poverty and inequality on health	30	47%
International comparison of health systems	29	45%
Globalization and health	28	44%
International comparison of burden of disease	28	44%
Tropical medicine	28	44%
Travel medicine	19	30%
International health and development	16	25%
International movement of people	14	22%

This survey therefore suggests that the scope of global health education is diversifying beyond a concern with tropical medicine. The emerging interest in globalization may indicate that medical schools in different parts of the world are also evolving away from the ‘us’ and ‘them’ concerns of international health. The traditional concerns of the international health era are still present, with comparative teaching on the characteristics of different national health systems and burden of disease. Determinants of health were also high on the agenda of those responding to the survey, as reflected in the relatively large number of schools teaching on international perspectives on poverty and inequality and their effects on health.

The majority of teaching in global health topics was carried out either by biomedical lecturers alone (64%) or jointly with non-biomedical lecturers (29%). Sole non-biomedical teaching was relatively rare (7%). This is likely to reflect the medical school setting of teaching delivery. Encouragingly, ‘globalization and health’ and ‘international comparison of disease burden’ were the topics most regularly taught by a combined team.

#### Training and career paths

In light of these evolving trends, we now consider how global health education for undergraduate medical students might be structured in the future and how this may align with postgraduate teaching. This involves thinking about what kinds of doctors are needed in the global health workforce. It should be noted, however, that we believe global health teaching is essential for all medical graduates. This section focuses only on those who choose global health as a career path.

We set out three categories of global health doctor that are different from the ‘international health’ concerns of tropical medicine and infectious diseases (Table 3).

**Table 3:** Schematic representation of curricula for global health

**Intervention points**	**‘Globalised doctor’**	**‘Humanitarian doctor’**	**‘Policy doctor’**
Core curricula	√	√	√
Specialised developing countries medical track		√	
Optional intercalated degree	√	√	√
Optional student selected component / elective course	√	√	√
Overseas clinical placement	√	√	√
Postgraduate study (MSc/MPH)		√	√
Postgraduate study (DTM&H)		√	

The first of these categories is the ‘globalised doctor’. Globalization has altered the context of medical careers, by creating new opportunities for medical migration, shifting the cultural profile of patient populations, facilitating global pandemics, and changing the regulatory environment for health systems. All UK doctors (indeed doctors trained to work within any ‘national’ health system) therefore need exposure to some aspects of global health teaching. Medical students and doctors are also increasingly involved in global health advocacy, and project links with developing countries. These initiatives highlight an enthusiasm to introduce aspects of global health into medical training and with it provide an opportunity to teach students about public health and its broader determinants in a context that interests them.

The second category is the ‘humanitarian doctor’. This is the traditional route for those concerned with health in other parts of the world and who want to take a hands-on approach to medicine elsewhere. This is now facilitated in some UK hospitals through formal links arranged by organisations such as the Tropical Health and Education Trust [21]. For doctors from the developed world, there is a need for special training usually conducted at postgraduate level in the aetiology and treatment of diseases common in poorer countries, but it is acknowledged that these doctors also need training in the social aspects of health and healthcare [22].

The final category is the ‘policy doctor’. This is perhaps the smallest and least well-recognised category of medical careers in global health. Yet doctors populate (and often dominate) institutions of global health research, aid and governance as well decision-making posts in national Ministries of Health [23]. The policy arena thus stretches over a wide array of institutions, including intergovernmental bodies such as the WHO and the European Union’s Directorates General Development and Cooperation, Health and Consumers, and Humanitarian Aid, Global Public-Private Partnerships, such as the Global Alliance for Vaccines and Immunization (GAVI), and philanthropic research and implementation bodies such as the Bill and Melinda Gates Foundation and the Wellcome Trust. Additionally, the increasing role of General Practitioners in commissioning of healthcare proposed in the UK’s Health and Social Care Bill [24] means that the changing skill set required of doctors is not just in global health. Concerns have been raised that the domination of traditionally-trained doctors in policy-making can lead to an inappropriate medicalisation of policy, and a neglect of broader interventions to improve global health [23]. This re-emphasises the need for early introduction to cross-disciplinary perspectives in the teaching of global health in medical curricula.

Table 3 also sets out a number of intervention points for training in global health for each of these three types of doctor. These reflect the different options available for doctors in high income countries and specifically the UK. We recognise that doctors may shift across these boundaries in the course of their careers, but the purpose here is to delineate the type of global health training predominantly needed in each category. This builds on the principles of global health we outlined in the accompanying paper, which emphasised multi-disciplinarity, a need for the introduction of a range of perspectives on global health, and critical discussion of interventions and values. The extent and intensity of engagement with these principles will vary depending on the points of intervention in curricula, which we now discuss. However, the multiplicity of the potential points of intervention is also useful in helping to meet some of the demands for reflexivity rather than didactic teaching of competencies; as well the demand to teach about local specificity rather than simply commonalities between countries.

### Points of intervention

#### Core curricula

We have attempted to justify why all medical students need some training in global health. In fact, in the UK, many of the themes we have discussed as key to global health training are already touched on (for example cultural and social issues within professional development courses and medical sociology). Many are also currently taught from a national perspective but have the potential to be globalised (public health teaching on the social determinants of health for example). Since the first edition of *Tomorrow’s Doctors*, the General Medical Council has prescribed teaching on the broader social context of health for medical curricula. A recent review of *Tomorrow’s Doctors* has also highlighted the need for teaching on the structures of the UK National Health Service (NHS) [19] and a recent article in the Lancet outlined the topic areas that the authors argue need to be included in all UK undergraduate medical curricula [25].

Our own observations and those of a recent study of German medical students [7] suggest that there is a widespread lack in popularity of population health elements of medical training among medical students, which contrasts with the enthusiasm for more global health teaching that we have described above. This paradox suggests that there is a potential to create more enthusiasm for public health issues already in curricula by adding a global dimension. Furthermore, making global issues a routine part of medical training would be a good way of encouraging students to work in other parts of the world when they have qualified.

Subjects that might be fruitfully addressed by adding a global health dimension to current teaching include worldwide patterns of health and disease; the up- and downstream determinants of these patterns; globalization and its effects on health and health systems; and a comparative study of the student’s own national health system with other health systems. Teaching in core curricula could therefore both *engage* students by noting the common points of interest between national and global health and *stretch* their aspirations (of where they will work in the future for example), by pointing out some of the differences in circumstances in other parts of the world. Reflection on values and perspectives could also be encouraged by engagement with issues of cultural competency and addressing the needs of diverse population groups. Learning could take place either through public health teaching or through a Professional Development vertical/ongoing curriculum track.

#### Special overseas doctor track

The idea of adding a special track to medical training specifically for students who have a strong desire to work for part of their career in overseas health systems is innovative. Such schemes could be divided by region or by the level of development of the health system in question. Therefore, in addition to standard medical training, a developing country stream might include tropical medicine, the social context of health and disease across the world, research and project management skills, language skills, reflection on attitudes (cultural competency) and regular exposure opportunities in poor countries. A European stream could be based on the current European Option in the Manchester University MBBS programme, which gives students the opportunity to acquire language skills and spend periods of time studying in Europe. While such tracks are increasingly popular in postgraduate training ^a^, they are less so at undergraduate level.

A similar overseas doctor track would appeal to students who have an interest in learning about the specific health needs of other countries, with a curriculum focused in that direction. Clearly medical schools need to carefully weigh demand before making heavy investments in such initiatives. Although many students qualifying from this track would eventually choose to spend their working lives in the UK, they would have gained valuable skills that would prepare them for diverse careers.

#### Optional student selected components

In British medical schools, Student Selected Components (SSCs) are core aspects of the medical degree but which allow students a choice of modules and therefore the freedom to explore issues of particular interest. According to the UK’s General Medical Council [19], Student Selected Components (SSCs) must be part of curricula and should be used to enhance professional and personal skills and, in later years, explore career opportunities. Currently, several British medical schools run SSCs on global health covering a wide range of subjects [18]. All types of global health doctor are served by the promotion of these optional modules and their proliferation should be encouraged. Again, they may play the role of incentivising ‘mainstream’ medical students to consider a global health future of some kind, but also (given the small group format in which they are generally run) be an ideal space for reflexive thinking.

#### Elective programmes or international rotations

Elective programmes (or as they are known in North America, International Rotations or clerkships) for medical students are well established in developed countries offering widespread opportunities, and usually encompass a six to ten week exposure to medicine in another country. In the UK approximately 40% of medical students visit a developing country during their elective period [2]. The US and Canada numbers are similar with about a third of students participating in a global health rotation [26], while in Germany 36% of students travel to developing countries as part of their course. [7] Published studies of the elective experience provide evidence that it leads to improvements in understanding of tropical diseases, cultural awareness, health systems and public health [27]. Enhanced skills resulting from overseas rotations have included improvements in problem-solving, clinical examination, laboratory expertise and linguistic ability; in addition, changes in student values have been observed with a particular emphasis on showing more of a disposition to serve the excluded [27]. Others have suggested that electives may help students to gain a sense of ‘idealism’ and that they may serve as a buffer against the declining interest in primary care and in working with underserved communities which is typical in the later training years [28]. At UCL, a combination of the elective period with a month long Student Selected Module in global health provides an introduction to the issues with which the student is likely to be confronted during the elective [2].

Considerable selection bias and a lack of objective measurement of outcomes limit the strength of studies about the elective period [29]. Nevertheless these types of ‘immersion’ experience in circumstances radically different from one’s own are recognised to be of value in other fields [30]. Encouraging medical students to participate in them would seem to be of value in creating the kinds of global health doctors we have outlined above. In recognition of the way electives are often focused on experiential rather than guided learning [31], some institutions, for example in the UK, Netherlands and Canada [2,4,8], have tried to provide a more structured environment for student learning. This generally combines the overseas experience with a block of teaching before, during or after the elective. Programmes teach a range of issues related to global health and also encourage students to complete projects as part of their fieldwork in the developing country. Programmes at UCL and in Finland have also incorporated an exchange with students from medical schools in resource-poor countries to facilitate joint study and peer learning [2,6]. Further emphasis on allowing students to reflect on the differences they have observed between countries’ health systems and people’s health behaviours seems to be a central element of rounding off the elective opportunity.

#### Optional intercalated degrees

Several UK medical schools offer opportunities for intercalation of a one year full-time Bachelor of Science (BSc) and, more recently, Master of Science (MSc) as part of medical training. UCL established the first BSc in international health in 2001 [18], and since then seven other UK medical schools have joined them. Existing intercalated degrees have incorporated a wide range of topics, although there are differences in emphasis between courses. For example, UCL has a focus on exploring policy issues and the determinants of health worldwide [18] whilst Leeds focuses on health research and health systems mainly in developing countries [32]. These differences are likely to reflect both differences in loci of expertise and in the evolving definitions of global health outlined earlier. Intercalated degrees are useful for those wishing to pursue policy, research or humanitarian careers, giving students a solid basis for postgraduate study and highlighting potential career opportunities. The differences between each programme are healthy from the point of view of student choice.

We have recently surveyed 120 Alumni from the first 6 years of the UCL Intercalated BSc in Global Health (2001–02 to 2006–07), with a 69% response rate [33]. Ten of the respondents (12%) were training in public health compared to a national figure of 0.8% of UK graduates [34] and another 22 (27%) were involved with public health as part of their job. Forty two Alumni (51%) were involved in advocacy either in or outside their work, and 32 (39%) undertook some volunteering. Of those responding, 32 (39%) already had undertaken some work overseas, 18 in Africa, 4 in Latin America and 2 in Asia, while 49 respondents (59%) definitely planned to work overseas in the future. Two of the Alumni were working as Teaching Fellows in Global Health and two others had also done so; one was working for the UK Department for International Development. It is clearly impossible to attribute to the BSc course any of the career choices, work and non-work activities, or overseas work intentions, because of the strong possibility of pre-selection. It is nevertheless encouraging that committed and enthusiastic students with a global orientation and outlook were provided with the knowledge, understanding and skills to enable them to transform commitment into action.

#### Postgraduate courses MSc/MPH/DTM

Following graduation, it is possible for doctors to build upon their undergraduate training with postgraduate courses, better equipping them for their role as either a ‘humanitarian’ or ‘policy’ doctor. As well as the integrated postgraduate training programmes mentioned above, a variety of courses are available either at Masters level in a range of global health related subjects, or as Diplomas in Tropical Medicine, and are key qualifications for doctors wishing to work in humanitarian, research or policy arenas. We do not discuss them further here, except to note that Masters programmes too, appear to be evolving away from a general core concentrating on health economics, behavioural science and epidemiology, towards considering broader political, economic and cultural issues in developing countries, if not the whole world [35].

## Summary

In this article we have outlined how global health education might respond to the changing understandings of global health. In particular we outline changes that have taken place in UK medical curricula – and through a snapshot survey, worldwide – towards teaching that can be defined as ‘global’ rather than ‘international’ health. The key point to consider is the fact that the movement towards global health will inevitably require greater input from disciplines outside medicine and public health. While there is still demand for training in specialised areas such as tropical medicine, in the future we believe that the three types of global health doctor we have outlined here – the globalised doctor who is primarily based within their own health system, the humanitarian doctor and the policy doctor – will all become recognised and valid categories, so predicating training in the broader social, economic and political aspects of global health. This must be complemented by critical reflection on the perspectives and values that underlie ‘global health’ as a necessary part of students’ own professional development, whether they continue to work at home or abroad.

## Endnotes

^a^See for example: 1. GHE/IM Residency Boston [Internet]: Brigham and Women’s Hospital [cited 2010 June 2]. Available from: http://www.brighamandwomens.org/socialmedicine/gheresidency.aspx and 2. Tropical Training [Internet]. Amsterdam: Netherlands Society for Tropical Medicine and International [cited 2010 June 2]. Available from: http://www.nvtg.org/index.php?id=136.

## Abbreviations

BSc: Bachelor of Science; FAIMER: Foundation for Advancement of International Medical Education and Research; GAVI: Global Alliance on Vaccines and Immunizations; GMC: UK General Medical Council; MSc: Master of Science; NHS: UK National Health Service; SSC: Student Selected Component; UCL: University College London.

## Competing interests

The authors declare that they have no competing interests.

## Authors' contributions

MR led the development of the framework for the paper, wrote the first draft of the paper, commissioned and helped shape the survey instrument, helped analyse results and co-ordinated the final rewrite of the paper. AS commented on initial drafts, helped shape the survey instrument, implemented the survey, helped analyse the results and co-ordinated the final rewrite of the paper. RH commented on initial drafts, helped shape the survey instrument and contributed to the final rewrite of the paper. OJ contributed to the final rewrite of the paper. AM commented on initial drafts and contributed to the final rewrite of the paper. SM contributed to the initial drafts of the paper. FM contributed to the final rewrite of the paper. JJM contributed to the final rewrite of the paper. VP commented on initial drafts, helped shape and implement the survey instrument, helped analyse the results and contributed to the final rewrite of the paper. RW commented on initial drafts and contributed to the final rewrite of the paper. CW commented on initial drafts and contributed to the final rewrite of the paper. JSY commented on initial drafts and contributed to the final rewrite of the paper. All authors read and approved the final manuscript.

Note: the findings, interpretations and conclusions expressed in this article are those of the authors and do not necessarily reflect the views of their employers.
